# An adult spindle cell rhabdomyosarcoma in the head and neck region with long-term survival: a case report

**DOI:** 10.1186/1752-1947-8-208

**Published:** 2014-06-19

**Authors:** Stefan Hartmann, Grit Lessner, Thomas Mentzel, Alexander C Kübler, Urs DA Müller-Richter

**Affiliations:** 1 Department of Oral and Maxillofacial Plastic Surgery, University Hospital Würzburg, Pleicherwall 2, 97070 Würzburg, Germany; 2 Institute of Pathology, University Würzburg, Josef-Schneider-Straße 2, 97080 Würzburg, Germany; 3 Dermatopathologische Gemeinschaftspraxis, Siemensstraße 6/1, 88048 Friedrichshafen, Germany

**Keywords:** Rhabdomyosarcoma, Spindle cell, Adult, Surgery, Head

## Abstract

**Introduction:**

Spindle cell rhabdomyosarcoma of the head and neck is a very rare tumor in adults. We report on one case with long-term survival.

**Case presentation:**

A 41-year-old nonsmoking Caucasian man presented in June 2007 with a painless swelling under his tongue. A diagnosis of a soft tissue sarcoma, and a myofibrosarcoma in particular, was made via biopsy. After multimodal treatment, including local and systemic therapy, our patient remained disease-free until September 2010. The local recurrence was treated unsuccessfully with various chemotherapy regimens. In September 2011, our patient underwent surgical resection again, and a spindle cell rhabdomyosarcoma was diagnosed. To analyze the mismatch between the original diagnosis of a myofibrosarcoma and the second diagnosis, the two specimens were reassessed, and a final diagnosis of a spindle cell rhabdomyosarcoma was made. In 2012 and 2013, our patient suffered further recurrences that were surgically treated, and he is still alive with disease six years and 10 months after the initial diagnosis in June 2007.

**Conclusions:**

In adults, the spindle cell rhabdomyosarcoma tumor is very rare in the head and neck region. In contrast to childhood tumors, spindle cell rhabdomyosarcoma in adulthood is often associated with a poor prognosis. In the present case, the radical surgical treatment might have helped to prolong the patient’s overall survival, which has lasted more than six years. To our knowledge, this is the longest overall survival reported so far for this tumor entity in the head and neck region.

## Introduction

Most malignancies of the oral cavity are squamous cell carcinomas. This tumor entity is strongly related to several risk factors, such as tobacco and alcohol use [1]. Sarcomas of the head and neck region are very rare tumors that are only found in approximately 1% of all head and neck malignancies [2,3]. In particular, spindle cell rhabdomyosarcoma (sc-rms) was first described in the pediatric population [4]. The occurrence of sc-rms in adults was first reported by Rubin *et al*., whereas Mentzel *et al*. described sclerosing pseudovascular rhabdomyosarcoma, an additional morphological variant of sc-rms in adults [5,6]. In contrast to sc-rms in childhood, sc-rms tumors are very aggressive and associated with a poor prognosis in adults [7]. In further contrast to sc-rms in childhood and adolescence, when the genitourinary tract and the orbital site are most commonly affected, the location of sc-rms in adults is mainly the head and neck region, except for the orbital site [8,9], and the deep soft tissue of the extremities [10,11]. Furthermore, Esnaola and colleagues showed that common predictors of survival, such as location, nodal status and histological subtype, are not useful for predicting rhabdomyosarcomas in general. The key factors for predicting overall survival are more likely metastatic disease at presentation, a poor response to chemotherapy, tumor size and a negative margin status after primary resection [8,10,12]. The impact of age on survival is still a controversial subject [8,10,12]. Although the data available on the prognostic effects of genetic characteristics is still limited, the *PAX3-FOXO1* fusion gene and the *PAX7-FOXO1* fusion gene, which are associated with alveolar rhabdomyosarcoma, seem to be related with a worse prognosis because of earlier metastatic spread [13,14]. However, no distinct genetic signature has been reported for sc-rms thus far, although certain cases feature a loss of heterozygosity in chromosome 11p15.5 [15].

Because of the very low incidence of these tumors, clinical knowledge of head and neck rhabdomyosarcoma treatment in adults is very poor, and this treatment leads to unsatisfying results (Table 1).

**Table 1:** Short overview of the survival rate of similar cases reported in the past

**Age (years)**	**Sex**	**Follow-up (month)**	**References**
49	F	Died of therapeutic complications (1)	[11]
51	M	Alive without evidence of disease (48)	[7]
38	F	Died of disease (27)	[9]
21	F	Alive without evidence of disease (24)	[9]
38	M	No evidence of disease (1)	[9]
18	M	Alive with disease (17)	[9]
22	M	Alive with disease (16)	[9]
26	F	Alive without evidence of disease (12)	[9]
18	F	Died of disease (20)	[16]
19	M	Died of disease (8)	[16]
33	F	No evidence of disease (1)	[17]

F, female; M, male.

## Case presentation

**Figure 1:**
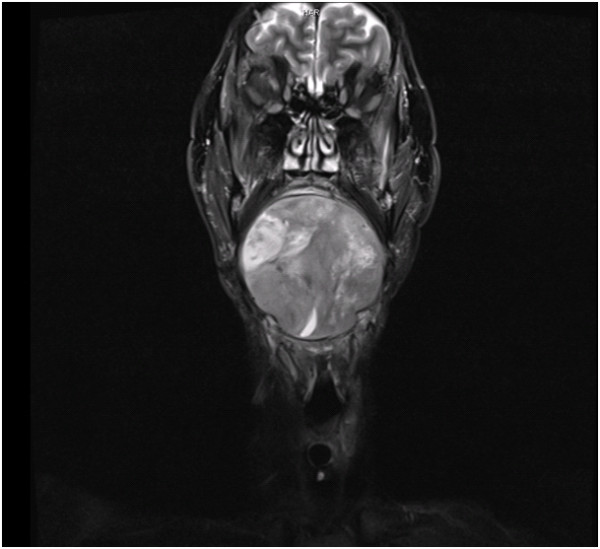
**A preoperative magnetic resonance imaging scan in August 2011.** The entire floor of the mouth, including the tongue, is filled by the tumor.

**Figure 2:**
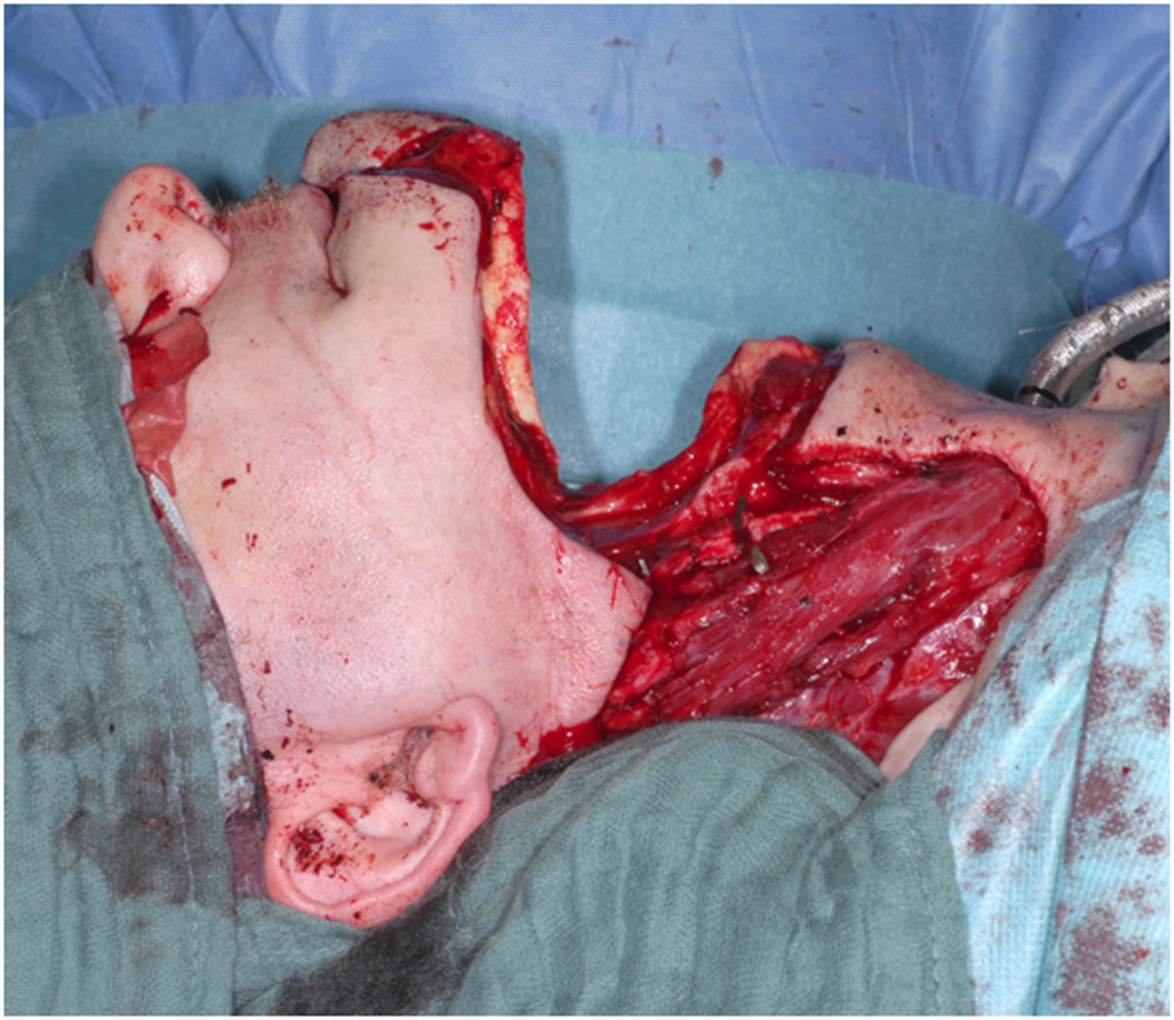
Intraoperative situs after temporary mandibulotomy and tumor resection.

A 41-year-old nonsmoking Caucasian man presented in June 2007 with a painless swelling under his tongue, which was originally thought to be a sialadenitis of the sublingual or submandibular gland. Shortly afterward, a diagnosis of a soft tissue sarcoma, and a myofibrosarcoma in particular, was made via biopsy at another hospital. The following workup revealed no regional or distant spread. The chosen aggressive multimodal treatment was a modified version of a pediatric cooperative soft tissue protocol published in 2002 by the Cooperative Weichteil-Sarkomstudie (CWS) of the Society of Pediatric Oncology. Initially, our patient received three cycles of multiagent chemotherapy (vincristine, dactinomycin and ifosfamide), followed by radiographic restaging. Thereafter, our patient underwent surgical resection, including a radical neck dissection of levels I and IIa on both sides. The original tumor showed a size of 1.3cm in its greatest dimension and blurred edges, with a focal extension into the preepiglottic space margin (R1). Postoperatively, two more cycles of the same multidrug chemotherapy regimen were given in combination with external beam radiation (51 Gray (Gy)) to prevent local treatment failure. Until September 2010, our patient remained disease-free. He then developed local recurrence on the floor of his mouth, without signs of metastatic spread. Our patient was unsuccessfully treated with various chemotherapy regimens (paclitaxel + gemcitabine; Adriamycin® + ifosfamide; Adriamycin® + dimethyl-triazeno-imidazole-carboxamide (DTIC); and Yondelis® (trabectedin)). By August 2011, the tumor had reached a size of 11cm in its greatest dimension, which was the reason for a consult at our department (Oral and Maxillofacial Plastic Surgery) at the University Hospital of Würzburg (Figure 1). After restaging, our patient underwent total glossectomy with modified radical neck dissection on both sides. The epiglottis was removed, but the larynx was preserved. A wide resection of skin in the neck area was also necessary. Reconstruction of the several defects was performed with a latissimus dorsi free flap from the left side (Figure 2). The tumor was diagnosed as an adulthood sc-rms. To analyze the mismatch between the original diagnosis and the second diagnosis, we requested and reevaluated the slides of the original tumor specimen and accordingly corrected the former diagnosis of a myofibrosarcoma to a diagnosis of an adulthood sc-rms. Given negative surgical margins and the previously administered chemotherapies, our patient was discharged after three weeks in a good general condition, without any additional treatment beyond speech therapy. In September 2012, local recurrence in the anterior floor of the mouth was diagnosed and resected. Magnetic resonance imaging of the neck showed several suspicious lymph nodes in the nuchal area. Thereafter, a modified level III re-neck dissection (Robbins) was performed, and the locoregional disease was confirmed by histology. The reoperation was very well tolerated by our patient. This was again followed by a strict follow-up with close intervals. In May 2013, a third local recurrence was confirmed by biopsy. Further resection of the anterior floor of the mouth, parts of the latissimus dorsi flap, the sternocleidomastoid muscle, the right thyroid cartilage and the lateral pharyngeal wall had to be performed. Because of a pathological fracture of the anterior lower jaw that was caused by extensive tumor infiltration, parts of the lower jaw had to be removed as well, and continuity could not be obtained. To date, the patient is still alive with disease and is nourished via a percutaneous endoscopic gastrostomy (PEG) feeding tube.

**Figure 3:**
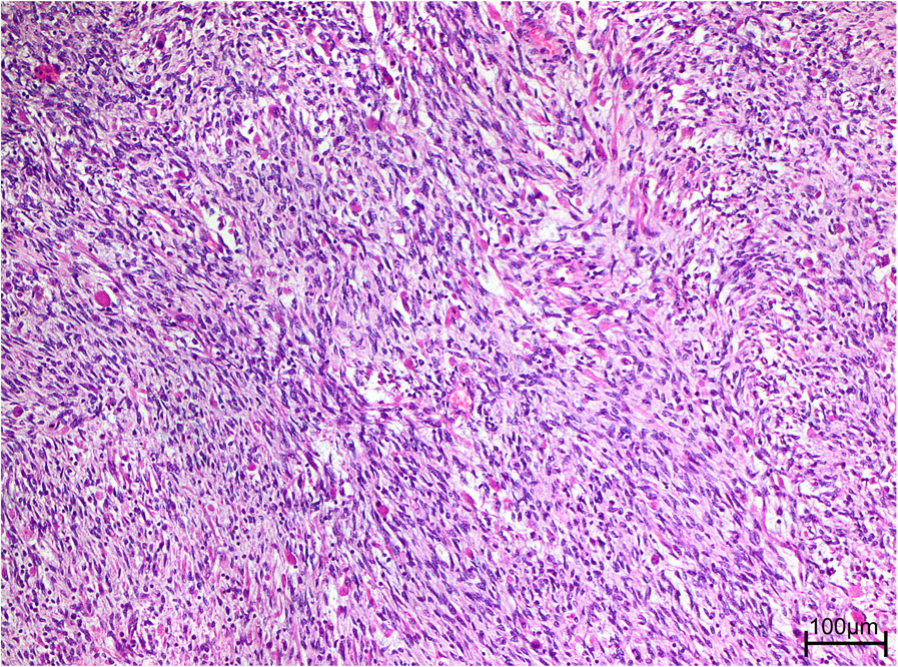
Rhabdomyoblasts are loosely scattered between the spindle cells (hematoxylin and eosin, original magnification x 100).

**Figure 4:**
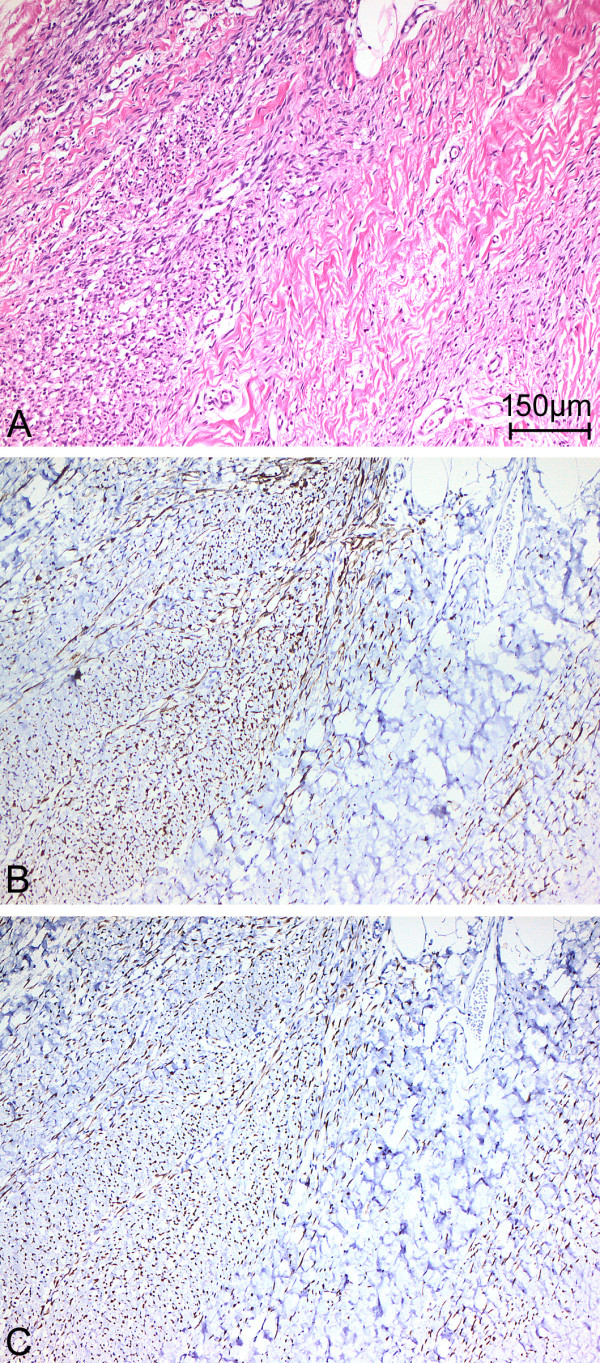
Distinctive morphology of the spindle cell rhabdomyosarcoma, as indicated by (A) hematoxylin and eosin staining and immunohistochemical staining for (B) desmin and (C) myogenic differentiation 1 (original magnification x 100).

**Figure 5:**
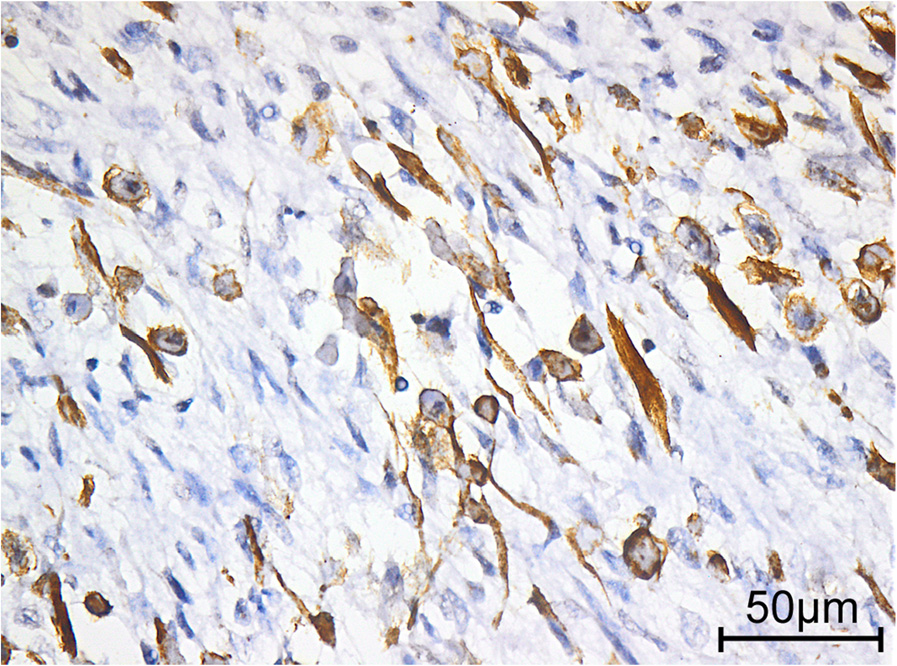
Immunohistochemical staining of desmin (original magnification x 40).

Macroscopically, the resection specimen of the first local recurrence (August, 2011) weighed 724g and contained a well-demarcated tumor measuring 11×8×8cm, without any invasion of the surrounding structures, including the extremely edematous tongue, epiglottis and skin. The cut surface of the tumor was white, firm and fleshy, without hemorrhage or necrosis. Microscopically, the tumor showed variable cellularity and was predominantly composed of spindle-shaped tumor cells with pale eosinophilic, partially ill-defined cytoplasm; an oval, tapered nucleus; vesicular chromatin; and mostly small eosinophilic nucleoli. In certain areas, morphologically, the tumor cells appeared more rounded and epithelioid. The tumor predominantly consisted of highly cellular areas characterized by an arrangement of long and intersecting fascicles of the neoplastic spindle cells, partially resembling a herringbone growth pattern. Occasionally, there were also regions with shorter fascicles of spindle cells surrounded by a collagen matrix. There was no sign of hyaline sclerosis of the intercellular matrix. Admixed throughout the tumor were a variable number of spindle-shaped and tadpole-like rhabdomyoblasts with abundant sparkling eosinophilic cytoplasm, an eccentrically placed nucleus and rare cross-striation. The rhabdomyoblasts were mostly loosely scattered between the spindle cells (Figure 3) and were not very obvious. However, there were also certain areas with lower cellularity and a conspicuous gathering of rhabdomyoblasts. Overall, the rhabdomyoblasts were the most significant morphological clue suggesting the correct diagnosis on conventional light microscopy [7]. Mitoses, including atypical forms, numbered up to 16 per 10 high-power fields (one high-power field = 0.53mm for the microscope used). Focal tumor necrosis was present, accounting for less than 1% of the total tumor volume. There was no obvious lymphangioinvasion or hemangioinvasion and no detectable invasion of the surrounding structures matching the gross description. All surgical resection margins were negative, as were all 52 lymph nodes.Immunohistochemically, the spindle cells partially expressed and the rhabdomyoblasts uniformly expressed desmin (Figures 4 and 5), whereas h-caldesmon staining remained negative. Furthermore, most rhabdomyoblasts and certain spindle cells showed specific nuclear positivity for the myogenic determination factors myogenin and MyoD1 (Figure 4). Actin exhibited focal positivity, with an accentuation at the periphery of the tumor. There was no immunoreactivity of the pan-cytokeratin marker KL1, and no S100, CDK4, MDM 2 or CD117 was detected.

In conclusion, the morphology and the immunophenotype led to the diagnosis of an sc-rms. Furthermore, these features indicated a tumor stage (TNM, 7th edition, 2010) of yrpT2b yrpN0 (0/52) L0 V0 Pn0, a resection status of R0 and a grade G3 tumor with six points in La Fédération Nationale des Centres de Lutte Contre le Cancer (FNCLCC) grading.

Additionally, we directly compared the morphology of the primary tumor specimen and our specimen from the first local recurrence, which showed the same histological features. To finally prove that these two specimens contained the same tumor entity, we complemented the primary immunohistochemical battery with tests for the specific markers of skeletal muscle differentiation myoglobin and MyoD1, which showed the same nuclear expression as described above. Therefore, a diagnosis of a sc-rms was made.

In the following resection specimens (2012 and 2013), including regional lymph nodes, the tumor tissue showed histology identical to that of the tumor described above, corresponding to further local recurrences and regional spread to lymph nodes, respectively.

## Discussion

The herein described case of an sc-rms of the head and neck region is very rare. The fact that the specimen was initially misinterpreted as a myofibrosarcoma emphasizes the difficulty in the histopathological diagnosis of these very uncommon entities. In this case, the herringbone growth pattern and the immunoreactivity of desmin and actin represent a diagnostic pitfall, resembling a myofibrosarcoma. However, the admixture of rhabdomyoblasts between the spindle cells and the expression of specific striated muscle markers led to the diagnosis of an sc-rms [15].

Of particular interest in this case is the patient’s overall survival of 82 months since his primary diagnosis in 2007, which is, to our knowledge, the longest overall survival of an adult patient with an sc-rms in the head and neck reported so far. When sc-rms was described for the first time in adults, Rubin *et al*. hypothesized a very poor outcome with short survival, which was modified by a larger case series by Nascimento and Fletcher in 2005 [6,9]. Additionally, this case is consistent with the observation that a good response to chemotherapy prolongs overall survival [12] and maximizes the metastasis-free period [8], which is related to a good prognosis. After the primary multimodal therapy, our patient had a disease-free survival period of 38 months, and he is still lacking distant metastases. Moreover, most adult patients affected with sc-rms suffer from a neoplasm of the head and neck region (56.25%) and are male (68.75%). The presented case is consistent with Nascimento’s study concerning sex, location and the possibility of long-term survival [9]. The poorer prognosis of adult sc-rms is in striking contrast to the prognosis of childhood sc-rms, which could be because older patients are less tolerant of intensive chemotherapy and that their tumors are less chemosensitive [8]. Furthermore, in adults, the location in the head and neck region might lead to less aggressive surgical treatment for esthetic reasons and limits the use of wide surgical margins [9]. The radical surgical treatment performed in the presented case could have helped to prolong the patient’s overall survival until today. This treatment might particularly be the reason for the patient’s long survival with disease because most patients rapidly die of the disease after recurrence [8]. Moreover, from a surgical perspective, it is important that tumor-free margins in a histopathological examination cannot predict the absence of local recurrence in the future. In particular, in the case of sarcoma, short follow-up intervals with radiographic control are necessary to detect locoregional recurrence.

## Conclusions

In cases of painless swelling in the head and neck region, rare differential diagnoses, such as sarcoma, should particularly be taken into consideration. In addition, an exact histopathological examination is needed because individually adapted therapy regimens are essential to properly treat affected patients. Despite aggressive surgical resection with free margins, local recurrence is common.

## Consent

Written informed consent was obtained from the patient for publication of this case report and any accompanying images. A copy of the written consent is available for review by the Editor-in-Chief of this journal.

## Abbreviations

CD117: cluster of differentiation 117; CDK4: cyclin-dependent kinase 4; DTIC: 5-(3,3-dimethyl-1-triazenyl) imidazole-4-carboxamide; Gy: Gray; KL1: katanin-like 1; MDM 2: mouse double minute 2 homolog; MyoD1: myogenic differentiation 1; *PAX3-FOXO1*: paired box 3-forkhead box protein O1; PEG: percutaneous endoscopic gastrostomy; rms: rhabdomyosarcoma; S100: S100 calcium-binding protein; sc: spindle cell.

## Competing interests

The authors certify that there is no conflict of interest with any financial organization regarding the material discussed in the manuscript.

## Authors’ contributions

SH and GL drafted the manuscript. GL and TM performed the pathohistological analysis. ACK, TM and UDAMR reviewed the manuscript. All authors read and approved the final manuscript.
